# Recent Advances in Polysaccharides from *Chaenomeles speciosa (Sweet)* Nakai.: Extraction, Purification, Structural Characteristics, Health Benefits, and Applications

**DOI:** 10.3390/molecules29132984

**Published:** 2024-06-23

**Authors:** Aiqi Yu, Wenjing Hu, Haizheng Bi, Lei Fu, Zhibin Wang, Meng Wang, Haixue Kuang

**Affiliations:** Key Laboratory of Basic and Application Research of Beiyao (Heilongjiang University of Chinese Medicine), Ministry of Education, Heilongjiang University of Chinese Medicine, Harbin 150400, China

**Keywords:** *Chaenomeles speciosa (Sweet)* Nakai., polysaccharides, structural characteristics, health benefits, structure–activity relationship, application

## Abstract

This article systematically reviews the extraction and purification methods, structural characteristics, structure–activity relationship, and health benefits of *C. speciosa* polysaccharides, and their potential application in food, medicine, functional products, and feed, in order to provide a useful reference for future research. *Chaenomeles speciosa (Sweet)* Nakai. has attracted the attention of health consumers and medical researchers as a traditional Chinese medicine with edible, medicinal, and nutritional benefits. According to this study, *C. speciosa* polysaccharides have significant health benefits, such as anti-diaetic, anti-inflammatory and analgesic, anti-tumor, and immunomodulatory effects. Researchers determined the molecular weight, structural characteristics, and monosaccharide composition and ratio of *C. speciosa* polysaccharides by water extraction and alcohol precipitation. This study will lay a solid foundation for further optimization of the extraction process of *C. speciosa* polysaccharides and the development of their products. As an active ingredient with high value, *C. speciosa* polysaccharides are worthy of further study and full development. *C. speciosa* polysaccharides should be further explored in the future, to innovate their extraction methods, enrich their types and biological activities, and lay a solid foundation for further research and development of products containing polysaccharides that are beneficial to the human body.

## 1. Introduction

**Figure 1:**
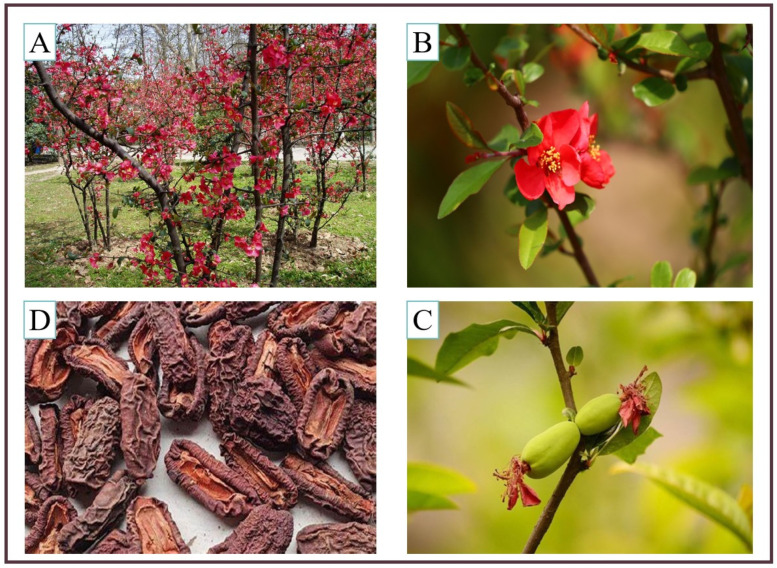
The morphological characteristics of *Chaenomeles speciosa (Sweet)* Nakai. (*C. speciosa*). (**A**) A plant of *C. speciosa*. (**B**) The flower of *C. speciosa*. (**C**) The fruit of *C. speciosa*. (**D**) The dried fruit of *C. speciosa*. (Pictures are from public sources and the Internet).

Natural plants are a huge treasure trove that can help alleviate human hunger and maintain health, and even fight disease [1]. Therefore, they are also known as nature’s gift to human beings. *Chaenomeles speciosa (Sweet)* Nakai. (*C. speciosa*) is a deciduous shrub plant belonging to the Rosaceae family [2]. The morphological characteristics of *C. speciosa* are shown in Figure 1. It has strong adaptability to climate and can tolerate semi-shaded, cold, and arid environments. It is mainly distributed in China, Korea, Japan, and other temperate climate countries [3,4]. Its fruit is a valuable source of healthy food due to its unique taste and rich nutrients such as sugars, proteins, fats, minerals, vitamins A and C, pectin, and malic acid [5]. Furthermore, its fruit contains a variety of natural active components and exhibits a wide range of health benefits, which are clinically used for the treatment of colds, asthma, hepatitis, and rheumatoid arthritis [6,7]. In addition, applying the sap of *C. speciosa* to ulcerated skin can alleviate ulcers and accelerate wound healing. It is worth mentioning that the effective components extracted from *C. speciosa* can be used to develop a new drug for the treatment of rare myofibrillar myopathy; this drug has been recognized as an orphan drug by the United States Food and Drug Administration (FDA). Therefore, it is also an important traditional Chinese medicine (TCM) and is included in the Pharmacopoeia of the People’s Republic of China (2020 edition) [8]. Furthermore, *C. speciosa* has a beautiful shape and bright colors, and also has certain ornamental value [9]. In short, *C. speciosa* is a plant with medicinal, edible, and ornamental values.

In recent years, the demand for health products has increased with the increase in people’s health awareness. The health value of *C. speciosa* has been paid great attention. *C. speciosa* is rich in polysaccharides, mineral elements, dietary fibers, amino acids, proteins, and other nutrients, and has high edible value [10,11]. In 2002, *C. speciosa* was included in the first batch of the homology list for medicine and food [12]. It can be used to cook delicious food or stew soup, with appetizing, beauty enhancing, and health promoting effects on the body. *C. speciosa* can also be made into preserves, jams, preserved fruits, fruit juice, etc., with a sweet and sour taste and a special fragrant fruit flavor. In addition, it is also a good raw material for brewing fruit wine and fruit vinegar. Therefore, the research and development of food and health products using *C. speciosa* has become a new trend.

Modern research has shown that *C. speciosa* contains a large number of bioactive components, such as polysaccharides, flavonoids, polyphenols, alkaloids, and volatile oils [13,14]. In recent years, with the development of analytical technology, macromolecular substances have become a popular research topic. Polysaccharides have attracted the attention of researchers due to their unique properties, including high biological activity and low toxicity [15,16,17]. They are providing a promising research platform for the development of new compounds, drugs, and functional foods [18,19]. Through the study of *C. speciosa* polysaccharides by researchers, it was found that *C. speciosa* polysaccharides have health benefits, including anti-diabetic, anti-inflammatory, analgesic, anti-tumor, immune regulation, and other effects, and can be widely used in the fields of food, medicine, cosmetics, and animal husbandry.

Due to their numerous medicinal and edible food qualities, *C. speciosa* polysaccharides have recently played a significant role in many fields and interest in research into their properties has increased. To the best of our knowledge, no updated systematic review of the plant’s polysaccharides has been published, which is required to help guide future *C. speciosa* polysaccharides research. In this study, the Chinese Pharmacopoeia, Flora of China, Web of Science, PubMed, and CNKI databases were searched within the last 13 years, using “*Chaenomeles speciosa* polysaccharides”, “*Chaenomeles speciose (Sweet)* Nakai. polysaccharides”, and “*C. speciosa* polysaccharides” as the key words, systematically reviewing the extraction and purification methods, structural characteristics, structure–activity relationship, health benefits, and applications of *C. speciosa* polysaccharides to provide insights for future studies. Therefore, this article presents a unique perspective on recent research into the extraction and purification methods, structural features, health benefits, and potential mechanisms of action of *C. speciosa* polysaccharides. Moreover, a complete analysis and discussion of the structure–activity relationship of *C. speciosa* polysaccharides is provided. Finally, the applications of *C. speciosa* polysaccharides are summarized. Overall, this review provides solid, scientific, and insightful information for the food and industrial applications of *C. speciosa* polysaccharides.

## 2. Extraction and Purification Methods of *C. speciosa* Polysaccharides

Extraction methods are pivotal in the research and product development of *C. speciosa* polysaccharides. There are various methods to obtain *C. speciosa* polysaccharides, although the main method is solvent extraction. The first consideration when using the solvent extraction method is to choose the appropriate solvent [20,21]. *C. speciosa* polysaccharides belong to polar macromolecules, and a solvent with stronger polarity should be selected as the extraction solvent. Existing research has shown that hot water has the characteristics of strong tissue penetration, high extraction efficiency, and low experimental cost, making it the most commonly used solvent for extracting *C. speciosa* polysaccharides. The extraction time of *C. speciosa* polysaccharide is generally 2–5 h, the number of extraction cycles is 2–3, and the solid–liquid ratio is 1:4, 1:10, 1:15, or 1:20 [22,23]. The differences in the above conditions are also important factors leading to the different extraction rates of polysaccharides from *C. speciosa*. The extraction solution obtained by this method may contain some insoluble substances, which can be removed by adding high concentrations (95%) of ethanol to the extraction solution because the polysaccharide is soluble in water and insoluble in ethanol [24]. The extraction solution can also be centrifuged to remove insoluble impurities [25]. After water extraction and alcohol precipitation, many impurities remain, such as inorganic salts, proteins, and small molecules. Therefore, in order to obtain high-purity polysaccharides, further separation and purification are needed. The use of dialysis can remove low-molecular-weight impurities [26]. The trichloroacetic acid method and Sevag method are commonly used to remove proteins [27]. A DEAE cellulose chromatography column can be used to remove pigments and achieve preliminary separation of polysaccharide components [28].

**Figure 2:**
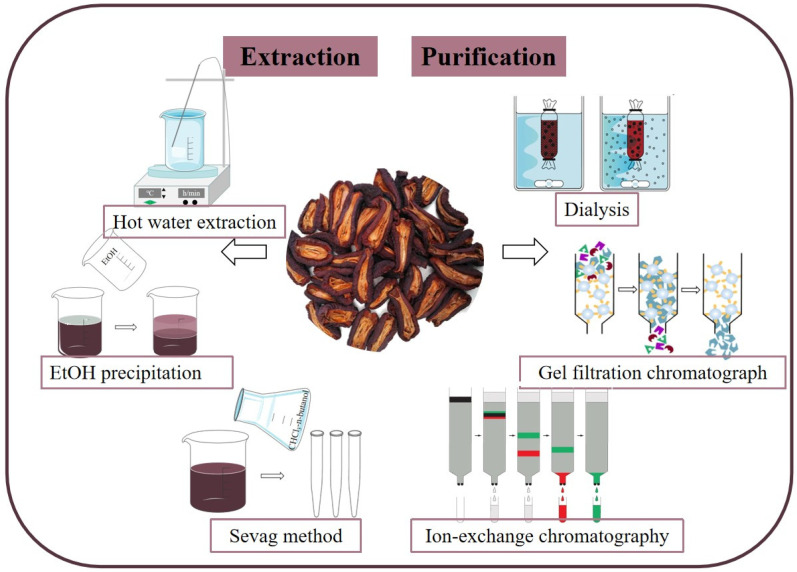
Extraction and purification of *C. speciosa* polysaccharides.

After extracting polysaccharides and removing non-polysaccharide components, crude *C. speciosa* polysaccharides can be obtained. However, these crude polysaccharides are a mixture that contains many polysaccharides with different molecular weights and structures [29,30]. Undeniably, production of highly purified polysaccharides will be conducive to precisely characterize their structures and understand the action mechanism of their bioactivities [31,32]. In order to obtain purified polysaccharides, the crude *C. speciosa* polysaccharides can be separated using column chromatography by selecting different materials [33,34,35]. For example, the purified polysaccharides F3 and CSP were obtained by DEAE-52 cellulose and Sephadex G-100 column chromatography, respectively [36,37]. In conclusion, the high-purity and high-activity polysaccharides can be obtained by selecting suitable extraction and purification methods, which provides a basis for further research and application. The extraction and purification processes of *C. speciosa* polysaccharides are shown in Figure 2.

## 3. Physiochemical and Structural Features of *C. speciosa* Polysaccharides

The structural characteristics are the basis for the biological activity of polysaccharides [38]. Currently, with the rapid development of isolation modern analytical technology and the deepening of *C. speciosa* polysaccharide research, nuclear magnetic resonance (NMR) spectroscopy, Fourier transform infrared spectroscopy (FT-IR) spectroscopy, high-performance gel permeation chromatography (HPGPC), high-performance liquid chromatography (HPLC), gas chromatography (GC), gas chromatography–mass spectrometry (GC–MS), scanning electron micrographs(SEM) morphology, methylation analysis, acid–base degradation, and other biological means are widely used in the study and identification of *C. speciosa* polysaccharide structures [39,40]. So far, a variety of polysaccharides have been identified from *C. speciosa*, and their monosaccharide composition, molecular weight, and structural characteristics are shown in Table 1. In addition, the table also includes their names and corresponding references.

### 3.1. Monosaccharide Compositions

Monosaccharides are the basic units and components of polysaccharides [41,42]. *C. speciosa* polysaccharides are first hydrolyzed into monosaccharides by acid hydrolysis. Compared with standard monosaccharides, the hydrolyzed products are derived and then analyzed qualitatively and quantitatively by GC or HPLC. A polysaccharide (F3) was isolated from *C. speciosa* seeds, and HPLC analysis showed that F3 is an acid polysaccharide mainly composed of Rha, GlcA, Gal, and Ara, with a molecular molar ratio of 6.34:5.73:47.14:40.13 [36]. CSP-W-2 polysaccharide was isolated from *C. speciosa* fruit, and its monosaccharide composition was analyzed by GC-MS [43]. The results showed that CSP-W-2 is a neutral polysaccharide, mainly composed of Glc, Gal, Ara, Man, and Xyl, with a molecular molar ratio of 3.7:3.2:1.7:0.9:0.4. It can be seen that the monosaccharide compositions and ratios of polysaccharides extracted from different parts are different [44]. CSP-h is a *C. speciosa* polysaccharide composed of Man, Rha, GalA, Glc, Gal, and Ara [45]. HPLC is the most commonly used method for analyzing monosaccharide compositions [46]. In general, *C. speciosa* polysaccharides are rich in composition, including glucose (Glc), galactose (Gal), rhamnose (Rha), xylose (Xyl), mannose (Man), arabinose (Ara), glucuronic acid (GlcA), and galacturonic acid (GalA). Among these, GlcA and GalA are only present in a few *C. speciosa* polysaccharides.

### 3.2. Molecular Weights

Molecular weight (MW) is also considered to be a crucial factor in the structure characterization of *C. speciosa* polysaccharides. Common methods used to determine the molecular weights of polysaccharides include HPGPC, vapor osmotic pressure (VPO), viscosity, light scattering, mass spectrometry (MS), and chromatography [47,48]. Based on these techniques, the MWs of various *C. speciosa* polysaccharides mentioned and discussed in this paper are shown in Table 1. However, many factors, including the extraction site and experimental conditions, may have a significant impact on the MW of polysaccharides [49]. A polysaccharide F3 was obtained from *C. speciosa* seeds with an MW of 8.65 × 10^6^ Da [36]. The average molecular weight of the four polysaccharides obtained from *C. speciosa* fruit is 8.7 × 10^3^–8.65 × 10^6^ Da. In addition, Xie et al. used the hot water extraction (HWE) method with a solid–liquid ratio of 1:20 for 5 h. This operation was repeated twice to obtain two different polysaccharides (CSP and CSP-2) [23,37]. The molecular weight of CSP is 6.3 × 10^4^ Da, which is 1.4 times that of CSP-2.

### 3.3. Chemical Structures

**Figure 3:**
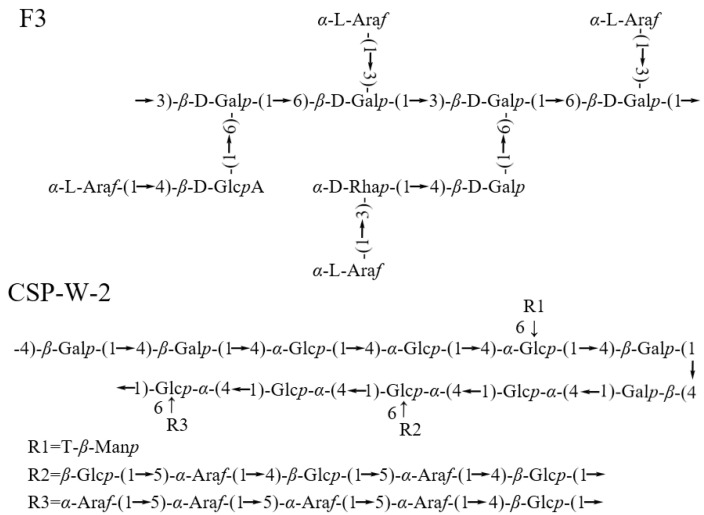
The chemical structure of *C. speciosa* polysaccharides.

Compared with the research on the monosaccharide components and molecular weight of *C. speciosa* polysaccharides in recent years, there is still a relative lack of published literature on the linkage types or conformational information of *C. speciosa* polysaccharides. The current inferred data on the repeat units of *C. speciosa* polysaccharides are shown in Figure 3. The results show that *C. speciosa* polysaccharides mainly contain neutral polysaccharides and acidic polysaccharides. From the information in Table 1 and Figure 3, it can be seen that CSP-W-2 is a neutral polysaccharide mainly composed of glucose and galactose (molar ratios of 37% and 32%, respectively), with the backbone of hexose, all in pyranose form (1,4 linked *β*-d-Gal*p*, 1,4 linked α-d-Glc*p*, 1,4 linked *β*-d-Glc*p*, and 1,4,6-*β*-d-Glc*p*) [43]. The precise structure of *C. speciosa* acidic polysaccharide F3 follows→3,6)-*β*-d-Gal*p*-(1→ is the backbone, branch is α-l-Ara*f*-(1→, →4)-*β*-d-Glc*p*A-(1→, →3)-α-d-Rha*p*-(1→, and→4)-*β*-d-Gal*p*-(1→ [36]. Despite these valuable insights, the precise structure and conformational details of *C. speciosa* polysaccharides remain elusive and diverse due to their inherent structural complexity, requiring further in-depth investigation.

**Table 1:** Extraction, purification, molecular weight, monosaccharide compositions, and structures of *C. speciosa*. polysaccharides.

Source	Polysaccharide Name	Solid Liquid Ratio	Extraction Time	Total Yield	Purification Method	Monosaccharide Composition	Molecular Weight	Structures	Ref
Fruits	CPS	1:4	2 h, repeated three times	0.31%	DEAE Sepharose Fast Flow column Gel permeation chromatography on Superdex 200 column	N/A	N/A	N/A	[22]
Fruits	CSP-2	1:20	5 h, repeat twice	5.28%	DEAE-Sepharose Sephadex G-100 column chromatography	Gal:Rha:Glc:Xyl = 3.8:1.6:1.2:0.4.	4.6 × 10^4^ Da	N/A	[23]
Seed	F3	1:10	N/A	5.72 ± 0.13%	Cellulose DEAE-52 column	Rha:GlcA:Gal:Ara = 6.34:5.73:47.14:40.13	8.65 × 10^6^ Da	The backbone of F3 was consisted of →3,6)-Gal*p*-(1→, and the side chains of F3 were composed of Ara*f*-(1→, →4)-Glc*p*A-(1→, →4)-Gal*p*-(1→ and →3)-Rha*p*-(1→.	[36]
Fruits	CSP	1:20	5 h, repeat twice	5.28%	DEAE-Sepharose Sephadex G-100 column chromatography	Glc:Gal:Rha:Ara = 4.6:1.3:0.8:0.5.	6.3 × 10^4^ Da	N/A	[37]
Fruits	CSP-W-2	1:4	2 h, repeated three times	4.1%	DEAE-Fast Flow column Superdex 200 column	Glc:Gal:Ara:Man:Xyl = 3.7:3.2:1.7:0.9:0.4	8.7 × 10^3^ Da	Its backbone is predominantly composed of 1,4 linked *β*-d-Gal*p*, 1,4 linked α-d-Glc*p*, 1,4 linked *β*-d-Glc*p*, and 1,4,6-*β*-d-Glc*p*, additionally some branches contained 1,5 linked α-l-Ara*f*, 1,4 linked *β*-d-Glcp, 1,3 linked α-l-Ara*f*, and T linked *β*-d-Man*p*.	[43]
Fruits	CSP-h	1:15	N/A	N/A	Deproteinization EtOH precipitation	Man:Rha:GalA:Glc:Gal:Ara = 1.66:2.92:4.72:4.25:9.42:77.02	N/A	N/A	[45]

N/A means not mentioned.

## 4. Health Benefits of *C. speciosa* Polysaccharides

**Figure 4:**
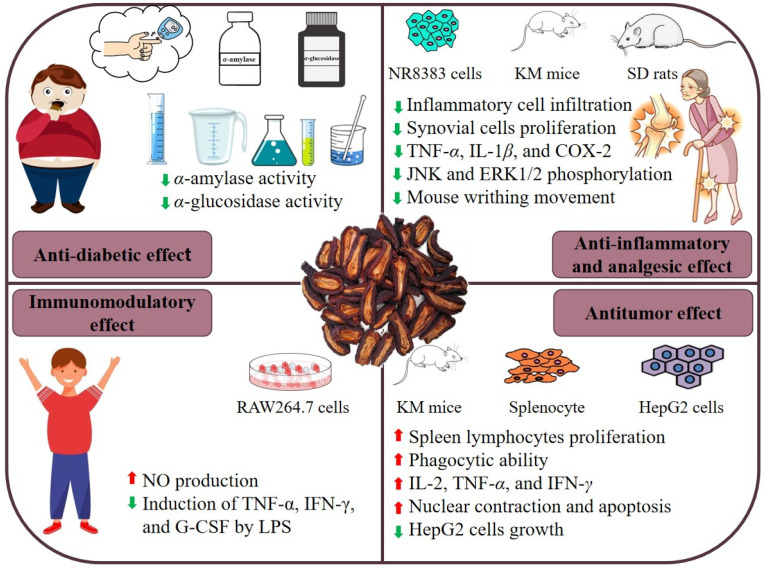
Health benefits of *C. speciosa* polysaccharides.

*C. speciosa* is not only a functional food but also a traditional Chinese medicine. Studies have shown that *C. speciosa* polysaccharides have significant biological activities, which have anti-diabetic, anti-inflammatory and analgesic, anti-tumor, and immunomodulatory effects. Table 2 provides information on the health benefits of *C. speciosa* polysaccharides, and the combined health benefits are shown in Figure 4.

### 4.1. Anti-Diabetic Effect

Diabetes is a public health issue, mainly divided into type 1 diabetes and type 2 diabetes [50]. Among these, type 2 diabetes, characterized by high concentrations of blood sugar, is considered one of the main economic burdens [51]. If left untreated, it can lead to serious complications, including hyperlipidemia and oxidative stress [52,53]. Therefore, the treatment of type 2 diabetes mainly focuses on reducing blood sugar fluctuations [54]. Anti-diabetic drugs have different possible means of action, one of which is to inhibit the enzymes required for polysaccharide digestion (these enzymes are called carbohydrases). Carbohydrases can inhibit or slow the absorption of carbohydrates, thereby lowering postprandial blood sugar levels [55,56]. One study determined the anti-diabetic properties of F3 by revealing its inhibitory activity against α-amylase and α-glucosidase. The key enzymes for starch and glycogen digestion are α-amylase and α-glucosidase, which play important roles in regulating glucose content. The research data show that F3 has good anti-diabetic effects. Compared with *C. speciosa* crude polysaccharides, F3 has higher α-amylase inhibitory activity and the lowest IC_50_ value, which is 6.24 mg/mL. However, the inhibitory ability of the positive control acarbose was significantly better than that of F3. Acarbose has a very good inhibitory effect on α-amylase and can also bring many side effects, such as bloating, diarrhea, and stomach pain, because undigested carbohydrates ferment in the intestine to produce gas, causing discomfort. F3’s relatively mediocre ability to inhibit α-amylase may result in fewer side effects than acarbose. In addition, the inhibitory activity of F3 on α-glucosidase is higher than that of crude polysaccharides and is concentration-dependent. Within the concentration range of 0.1–10.0 mg/mL, the inhibitory ability of F3 increases with its concentration. When the concentration reaches 10.0 mg/mL, the inhibitory activity of F3 on α-glucosidase is 72.65% higher than that of crude polysaccharides, which is 42.17%. Thus, F3 has great potential to be developed as an anti-diabetic drug [36].

### 4.2. Anti-Inflammatory and Analgesic Effects

**Figure 5:**
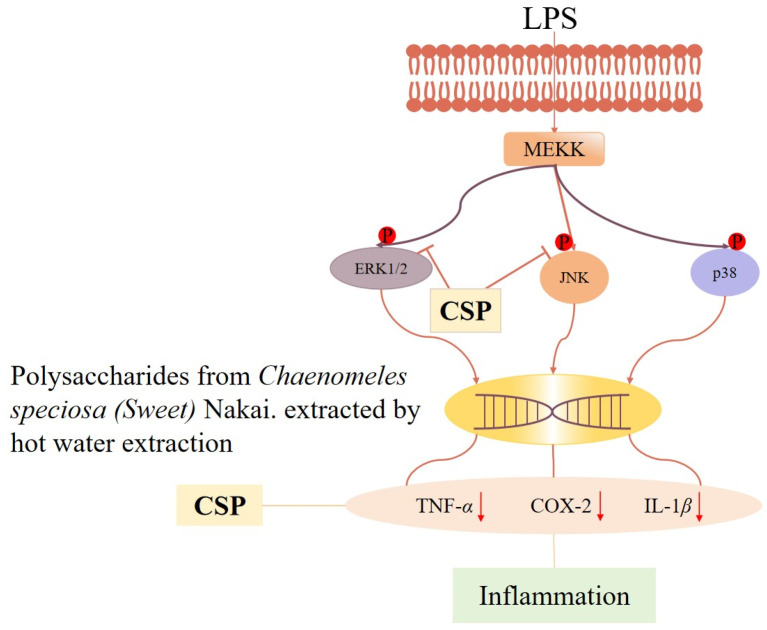
Mechanistic pathway diagram of anti-inflammatory and analgesic effects of *C. speciosa* polysaccharides. “↓” indicates decrease.

Inflammation is a defensive response of the body to stimuli, but long-term or excessive inflammation may lead to the development of diseases and tissue damage [57,58]. *C. speciosa* polysaccharides have anti-inflammatory activity and help alleviate inflammatory reactions. Research has found that *C. speciosa* polysaccharide (CSP-h) significantly reduces foot swelling, synovial tissue proliferation, and inflammatory cell infiltration in a complete Freund’s adjuvant (CFA)-induced Sprague–Dawley (SD) rat model of arthritis. Especially in the high- and medium-dose groups (25.0 and 50.0 mg/kg), CSP-h significantly improved foot swelling on day 22 after injection of CFA (*p* < 0.01), and the swelling inhibition rate in the high-dose group was similar to that of the positive control (irrecoxib). By observing the histopathology and quantifying the degree of pathological changes, it was found that there was less infiltration of inflammatory cells and less proliferation of synovial cells in the ankle joint of rats. Fibrosis of articular cartilage and bone tissue was not severe, and there was no significant narrowing of the articular surface [59]. Peripheral soft tissue adhesion and inflammation were not severe [60]. The number of pathological changes (moderate and severe) in the synovial and skin ulcers of rats in the CSP-h (50 mg/kg) group decreased by 33% and 67%, respectively, compared to the model group. Moreover, CSP-h was shown to inhibit pro-inflammatory cytokines (TNF-α, IL-1*β*, and COX-2) as well as JNK and ERK1/2 phosphorylation in LPS-stimulated NR8383 cells. Pain is another complication of inflammation, so the analgesic effect of *C. speciosa* polysaccharides has also been studied through mouse torsion experiments. The results showed that CSP-h dose-dependently promoted a decrease in mouse writhing movement. Especially in the high-dose group (100 mg/kg), it showed significant differences compared to the model group (*p* < 0.01). Therefore, the results of this study indicate that MAPK inhibition mediates the beneficial effects of CSP-h on hind paw swelling and HAC-induced twisting. Therefore, the secretion of pro-inflammatory cytokines and the downregulation of MAPK signaling promote the analgesic and anti-inflammatory effects of CSP-h (Figure 5) [45].

### 4.3. Anti-Tumor Effects

Cancer is one of the major diseases that threatens human health, and most chemotherapy drugs not only inhibit tumors but also cause significant damage to the human body [61,62]. TCM is highly valued due to its minimal side effects [63,64]. Existing research has shown that *C. speciosa* polysaccharides (CSPs) have anti-tumor effects. This experiment selected 50 mice, and all mice were subcutaneously inoculated with 0.2 mL of Sarcoma 180 (S180) cell suspension under sterile conditions. Then the mice were randomly divided into a positive control group (20 mg/kg cyclophosphamide), negative control group (physiological saline), and CSP high-, medium-, and low-dose groups (50, 100, and 200 mg/kg). Next, the tumor inhibition rate and spleen index of each group were compared. The results showed that CSP can inhibit the growth of transplanted S180 tumors in mice, especially at a high dose of 200 mg/kg, with a tumor inhibition rate of 44.9%. Meanwhile, CSP can also increase the relative spleen index of S180 tumor bearing mice. Compared with the negative control group, the CSP group had a significant increase in the spleen index (*p* < 0.05), while the cyclophosphamide (CTX) drug-positive group had a slight decrease in the spleen index. The spleen is one of the main organs in the body’s immune system, and the relative weight of the spleen is an important indicator of non-specific immunity, as it can reflect the development and immune function of immune organs. This suggests that CSP may inhibit tumor growth by strongly stimulating the immune function of S180 tumor-bearing mice. Lymphocyte proliferation is the most direct indicator of cellular immune function in the body. Subsequently, the immune regulatory mechanism of CSP on mouse splenic lymphocytes was explored at the cellular and molecular levels. By observing the effects of CSP on the proliferation of concanavalin A (ConA) and lipopolysaccharide (LPS)-induced splenocyte in vitro and the phagocytosis activity of peritoneal macrophages, it was found that CSP could significantly improve the proliferation of spleen lymphocytes in vitro within the range of 50–200 mg/kg, especially when the concentration of CSP was 200 mg/kg. The result was significantly different from that of the control group (*p* < 0.01). CSP treatment also has a promoting effect on the phagocytic ability of macrophages in S180 tumor-bearing mice. In addition, CSP treatment can improve delayed-type hypersensitivity (DTH) and promote the secretion of IL-2, TNF-α, and IFN-*γ* in the serum. In summary, the anti-tumor effect of CSP may be related to its effective immunostimulatory activity [37]. Additionally, the effect of *C. speciosa* polysaccharides on the growth of HepG2 cells was also explored. The *C. speciosa* polysaccharide CSP-W-2 can significantly inhibit the growth of HepG2 cells by enhancing nuclear contraction and apoptosis in a dose-dependent manner [43]. The above results indicate that *C. speciosa* polysaccharides may be a new source of natural anti-tumor products, with potential value in healthy foods.

### 4.4. Immunomodulatory Effect

The immune response, as an important physiological process, can recognize and destroy harmful substances or organisms from outside to prevent diseases, among which macrophages play an important role in phagocytosis, cytotoxicity, and intracellular killing activities [65,66]. Once activated, macrophages can directly resist pathogens through phagocytosis, or indirectly resist pathogens by producing related factors such as nitric oxide (NO), interleukin (IL), TNF-α, and reactive oxygen species (ROS) [67,68]. At present, plants with medicinal and edible value have received increasing attention due to their nutritional and medicinal value, as well as their bioactive components, especially their immunomodulatory activity, and *C. speciosa* is no exception [69]. A study has shown that CPSs have good immunomodulatory effects. In order to investigate the effect of CPSs on NO production, RAW264.7 cells were treated in the presence and absence of LPSs, respectively. No LPSs or CPSs were added to the control cells. According to the Griess reaction, the accumulated nitrite in the culture medium was measured as an indicator of NO production. The results showed that CPSs seemed to promote LPS-induced NO production in a dose-dependent manner. To test whether CPSs themselves can induce the production of NO, RAW264.7 cells were treated with a series of concentrations of CPSs and co-cultured without LPSs. The results showed that CPSs themselves can indeed induce the production of NO. Specifically, at 400 and 600 μg/mL, the NO production of CPS-treated cells was 2.5 times higher than that of the control group. In summary, these data indicate that CPSs themselves can induce the production of NO and act as an additive with LPSs. Furthermore, CPSs reduced the induction of TNF-α, IFN-*γ*, and G-CSF by LPSs. TNF-α are pro-inflammatory cytokines, participate in normal inflammatory and immune responses, and can synergistically regulate the production of other cytokines, cell survival, and cell death to coordinate tissue homeostasis. IFN-*γ* is a pro-inflammatory cytokine and an important activator of macrophages. Abnormal IFN-*γ* expression is associated with many autoimmune and inflammatory diseases. It has been determined that TNF-α and IFN-*γ* may serve as defense and regulatory molecules in inflammatory responses, with steady-state activity, including the production of nitric oxide in response to LPS stimulation. In addition, G-CSF is considered a regulator of immune or inflammatory responses [22]. Therefore, CPSs may pass through TNF-α, IFN-*γ*, G-CSF, and other pro-inflammatory factors to exert immunomodulatory effects.

## 5. Structure–Activity Relationship

In recent years, the research into polysaccharides has undergone a growing trend [70,71]. *C. speciosa* polysaccharides have been widely studied due to their various biological activities such as anti-tumor, anti-inflammatory, analgesic, and immune regulation effects. Numerous studies have shown that the structure of polysaccharides determines their activity, but there is currently no research summarizing the inevitable relationship between polysaccharide structure and its activity. Continuing to explore the relationship between polysaccharide structure and activity is of great significance for optimizing the application of polysaccharides in functional foods and drugs [72,73]. The structure of *C. speciosa* polysaccharides includes the composition, connectivity patterns, and branching characteristics of monosaccharides in polymer chains. These structural features significantly affect the biological activity of *C. speciosa* polysaccharides. Unfortunately, there is limited information on the correlation between the structure and biological activity of *C. speciosa* polysaccharides, as it is difficult to establish a direct relationship. Nevertheless, some previous studies have provided valuable insights into this relationship.

Accumulated data confirm that the biological activity of *C. speciosa* polysaccharides is influenced by their monosaccharide composition. These polysaccharides contain different monosaccharide units, such as Man, Rha, Ara, Glu, Gal, and Xyl, whose composition and proportion in the polymer chain affect their function [74]. For example, CSP and CSP-2 are *C. speciosa* polysaccharides isolated from the same raw material, with the former being crude polysaccharides and the latter being purified polysaccharides. CSP is composed of Glc, Gal, Rha, and Ara, exhibiting anti-tumor effects, while CSP-2’s monosaccharide composition is Gal, Rha, Glc, Xyl, and Gal, which have antioxidant effects [23,37]. In addition, the existing literature shows that polysaccharides with *α*-glucosidase inhibitory activity have some similarities in their monosaccharide composition ratios. *M. Charantia bioactive* polysaccharide (MCBP) is composed of Rha, Ara, Man, Gal, and GalA, and has strong *α*-glucosidase inhibitory ability [75]. Wang et al. reported polysaccharides from the fruits of wax apple, which have high levels of Gal and Glc and strong *α*-glucosidase inhibitory activity [76]. Furthermore, the proportion of Gal and Glc in *Coriolus versicolor* polysaccharides with hypoglycemic effects is relatively high, and experimental evidence has shown that high levels of Glc and Gal are important factors in inhibiting *α*-glucosidase [77]. Therefore, the mechanism of inhibiting *α*-glucosidase activity may be related to the binding of polysaccharides and enzymes [78]. This may lead to changes in the polarity and molecular conformation of *α*-glucosidase, resulting in partial loss of enzyme activity. F3 (*C. speciosa* polysaccharides) is composed of Rha, GlcA, Gal, and Ara in a molar ratio of 6.34:5.73:47.14:40.13, exhibiting excellent *α*-glucosidase inhibitory activity. The high content of Gal containing more hydroxyl groups and GlcA containing ketone groups in F3 is conducive to the binding of polysaccharides and enzymes. This can also explain why F3 has good glucosidase inhibitory activity [36]. The above studies indicate that monosaccharide composition is closely related to biological activity.

In addition to monosaccharide composition, the structure of polysaccharides is also an important factor affecting their activity [79]. Cheng et al. extracted a heteropolysaccharide CSP-W-2 from *C. speciosa* fruit, which contains Glc, Gal, Ara, Man, and Xyl. The main bonds of CSP-W-2 are 1,4 linked *β*-d-Galp, 1,4 linked *α*-d-Glc*p*, 1,4 linked *β*-d-Glc*p*, and 1,4,6-*β*-d-Glc*p*, together with the branches of 1,5 linked *α*-l-Ara*f*, 1,4 linked *β*-d-Glc*p*, D-Gal*p*, 1,4 linked *α*-d-Glc*p*, 1,4 linked *β*-d-Glc*p*, and 1,4,6-*β*-d-Glc*p*, and the branches of 1,5 linked *α*-l-Ara*f*, 1,4 linked *β*-d-Glc*p*, 1,3 linked *α*-l-Ara*f*, and T linked *β*-d-Man*p*. It can inhibit the growth of HepG2 by enhancing nuclear contraction and cell apoptosis and has anti-tumor potential [43]. According to the published literature, a purified polysaccharide (PPPF) from pumpkin fruit is composed of Gal, Man, Glc, and Ara. The main chain is composed of (1→6)-linked-Gal*p*, (1→6)-linked-Man*p*, and (1→3, 6)-linked-Man*p* with terminal branches (T-Glc*p* and T-Ara*f*) attached to O-3 of (1→3, 6)-linked-Man*p*. It can induce cell apoptosis by inhibiting the JAK2/STAT3 pathway in human liver cancer HepG2 cells [80]. Neutral polysaccharide (LGPS-1) was isolated from *Lentinus giganteus* and induced apoptosis of HepG2 cells through intrinsic mitochondrial apoptosis and the PI3K/Akt signaling pathway, demonstrating anti-tumor activity. Its monosaccharide composition is Man, Glc, and Gal. According to reports, the backbone of LGPS-1 was composed of 1,6-Gal*p* and 1,3,6-Man*p*, while the branches were composed of 1,6-Glc*p* and 1-Glc*p* [81]. Polysaccharides (PSPO01) with anti-tumor effects isolated from *Punica granatum* possess the *β*-1→3 Galp backbone as well as *β*-d Man*p* and *α*- d Man*p* side chains [82]. According to the above literature analysis, the hexose backbone, pyranose backbone, and neutral polysaccharides may play important roles in the anti-tumor activity of HepG2 cells.

In order to further understand the structural basis and exact mechanism of the biological effects of *C. speciosa* polysaccharides, it is crucial to study the molecular weight, chemical structure, chain conformation, and chemical modification of the *C. speciosa* polysaccharide structure. In the future, a large number of studies will be needed to identify the pharmacological mechanism and structure of *C. speciosa* polysaccharides in order to fully develop the application potential of *C. speciosa* polysaccharides and reduce the waste of resources.

## 6. Application of *C. speciosa* Polysaccharides

Consumers are increasingly interested in the health benefits provided by food ingredients. Fruit and vegetables deserve special attention as they are valuable sources of biological activity that determine the normal functioning of the human body [83,84]. *C. speciosa*, as a nutritious fruit, has also attracted the attention of researchers [85]. The polysaccharide components in *C. speciosa* have rich health benefits and are good raw materials for making functional foods [86]. Lozenges made mainly from *C. speciosa* polysaccharides have physiological functions such as anti-fatigue and antioxidant effects, and improve the body’s immune capacity. They have strong health benefits for the human body and are easy to carry and take. In addition, *C. speciosa* polysaccharides are also good feed additives. Additives composed of *C. speciosa* polysaccharides and other ingredients can replace the use of antibiotics in feed. This can not only effectively improve feed utilization and daily weight gain, but also improve the immune level of pigs, reduce the incidence rate of pigs, and promote the healthy growth of pigs. It has good market application prospects. *C. speciosa* polysaccharides can also be used to develop antiviral drugs. Research has shown that *C. speciosa* polysaccharides have a growth-promoting effect on poultry and can promote chicken growth. Specifically, after chickens are infected with the H7N9 avian influenza virus, *C. speciosa* polysaccharides can improve the immune function of chickens, which is beneficial for antiviral treatment, ensuring chicken health, promoting chicken growth, reducing breeding risks, and enhancing breeding efficiency. In addition, due to the unique physical and chemical properties and extensive biological activity of polysaccharides, various plant polysaccharides are widely used as health food ingredients or as the main components in food processing [87]. For example, Tremella polysaccharides can replace fat in low-fat ice cream, improving its water activity, swelling rate, and melt resistance, and can modify the nutrition of ice cream. It can be seen from this that polysaccharides have a wide range of uses. *C. speciosa*, as a medicinal and edible plant, has the characteristics of both food and medicine, but it is different from simple food or medicine. In terms of product development, it has broad prospects. The existing development of polysaccharide products can be referred to regarding their in-depth development.

## 7. Conclusions and Prospect

*C. speciosa* is a nutritious and beneficial fruit, as well as an important TCM [88]. In recent years, various extraction and purification methods have been used for *C. speciosa* polysaccharides, and their structures have been characterized by advanced technologies such as NMR, HPLC, and GC-MS. Biologically active substances found in plants may offer health benefits to humans [89]. As a bioactive substance, *C. speciosa* polysaccharides have been widely studied and shown various potential benefits, including anti-tumor, anti-inflammatory and analgesic, anti-diabetes, and immunomodulatory effects. In addition, the biological activity of *C. speciosa* polysaccharides is closely related to their structural characteristics. However, due to the complexity and diversity of these features, the relationship between them still needs further in-depth research. *C. speciosa* polysaccharides have the potential to serve as functional food or feed additives, but much exploration is still needed to achieve this potential. Maximizing the development of *C. speciosa* polysaccharides is a compelling vision for the future.

So far, a large number of studies have reported the extraction and purification of *C. speciosa* polysaccharides. However, there are still many challenges and problems that need to be solved. First, the exploration scope of extraction technology is too narrow and lacks innovation. Therefore, it is necessary to explore new extraction methods for *C. speciosa* polysaccharides, such as supercritical fluid extraction, subcritical water extraction, ultrasonic-assisted extraction, and enzyme-assisted extraction, which can be considered in the future [90,91]. In addition, in most cases, the first consideration when evaluating a suitable extraction method is the extraction rate, but attention has not been paid to the strength of polysaccharide activity. Determining how to improve the extraction rate while ensuring the activity of *C. speciosa* polysaccharide also needs to be studied. Second, impurity removal is one of the key steps in polysaccharide purification, so obtaining polysaccharides with a high yield and stable structure is one of the problems that need to be solved. Therefore, future research could choose resin adsorption, enzyme hydrolysis, freeze–thaw isotherms, and environmentally friendly methods to prepare purified *C. speciosa* polysaccharides [92]. Third, the research on the structure of *C. speciosa* polysaccharides mainly focuses on the primary structure, and there is relatively little research on the high-level structure. In order to study the advanced structure of *C. speciosa* polysaccharides, techniques such as atomic force microscopy, X-ray diffraction, and circular dichroism can be used as needed [93,94].

*C. speciosa* polysaccharides have multiple health benefits. However, most studies have only reported the in vivo or in vitro pharmacological activities of *C. speciosa* polysaccharides, their mechanisms of action lack in-depth research, and only animal experiments have been conducted. To realize the clinical application of *C. speciosa* polysaccharides, researchers need to make more efforts, and this is also the ultimate purpose of studying *C. speciosa* polysaccharides for practical production and clinical medicine. To accomplish this, researchers will need to blaze new trails. First, advanced technological means such as gene editing and proteomics can be used to reveal the targets and signaling pathways of *C. speciosa* polysaccharides and to further study their molecular mechanisms. Second, clinical research can be strengthened, larger and more rigorous clinical trials can be conducted, and the optimal medication and efficacy evaluation of *C. speciosa* polysaccharides can be explored. Third, the relationship between health benefits and structure still needs further research, and advanced technologies such as targeted mutagenesis or chemical modification can be used in the future to alter the structural characteristics of *C. speciosa* polysaccharides. This will provide valuable insights into the relationship between the structural characteristics and functional characteristics of these polysaccharides, thereby establishing their mechanisms of action and providing a comprehensive and in-depth basis for the widespread development of *C. speciosa* polysaccharides.

Polysaccharides are important components in *C. speciosa* that contribute to health benefits. However, there are few products that use *C. speciosa* polysaccharides as the main raw material, and the research and development of related functional foods, drugs, and cosmetics are clearly insufficient. The natural sources of plant polysaccharides have not been fully utilized, and they have lower toxicity, and thus have the advantage of promoting health. With the changes in people’s lifestyles and dietary structures, disease patterns have changed, and the number of unhealthy and chronic disease patients is increasing year by year. Determining how to prevent diseases, promote healthy lifestyles, and intervene has become a focus of people’s attention. The research and development of functional health foods using medicinal and edible plant polysaccharides as natural raw materials is in line with the health needs of contemporary consumers, and the combination of medicinal and edible TCM polysaccharides with food meets the needs of consumers for the nourishing, safe, and convenient characteristics of functional foods. Therefore, strengthening the product development of *C. speciosa* polysaccharides in functional foods is expected to bring new breakthroughs and progress to the application of *C. speciosa* polysaccharides. In summary, *C. speciosa* polysaccharides are highly potential active ingredients in *C. speciosa* and deserve further research.

## Figures and Tables

**Table 2:** Biological activities of *C. speciosa* polysaccharides and their mechanisms of action.

Biological Activities	Polysaccharide Name	In Vitro or In Vivo	Indicated Concentrations	Models/Test System	Action or Mechanism	Ref
Anti-diabetic effect	F3	In vitro	0–10.0 mg/mL	*α*-amylase and *α*-glucosidase	↓*α*-amylase and *α*-glucosidase activities The lowest value IC_50_ is 6.24 mg/mL	[36]
Anti-inflammatory effect	CSP-h	In vivo	12.5, 25.0 and 50.0 mg/kg	SD rats (120–140 g)	Improved foot swelling ↓Inflammatory cell infiltration ↓Synovial cells proliferation	[45]
		In vitro	12.5, 25.0 and 50.0 mg/kg	NR8383 cells	↓The expression of TNF-*α*, IL-1*β*, and COX-2 ↓JNK and ERK1/2 phosphorylation	
Analgesic effect	CSP-h	In vivo	25, 50 and 100 mg/kg	KM mice (23 ± 0.5 g)	↓Mouse writhing movement	
Anti-tumor effect	CSP	In vivo	50, 100 and 200 mg/kg	KM mice (20 ± 2) g	Improved spleen index	[37]
		In vitro	50–200 mg/kg	Splenocyte	↑The proliferation of spleen lymphocytes ↑The phagocytic ability of macrophages ↑The secretion of IL-2, TNF-*α*, and IFN-*γ* in the serum Improve delayed-type hypersensitivity	
	CSP-W-2	In vitro	0, 50, 100, 200, 400 μg/mL	HepG2 cells	↓The growth of HepG2 cells ↑Nuclear contraction and apoptosis	[43]
Immunomodulatory effect	CPS	In vitro	0, 100, 200, 400 and 600 μg/mL	RAW264.7 cells	↑The production of NO Reduced the induction of TNF-*α*, IFN-*γ*, and G-CSF	[22]

“↑” indicates increase; “↓” indicates decrease.

## Data Availability

No data were used for the research described in the article.

## References

[1] Pollier J., Moses T., Goossens A. (2011). Combinatorial biosynthesis in plants: A (p)review on its potential and future exploitation. Nat. Prod. Rep..

[2] Zhang S.Y., Han L.Y., Zhang H., Xin H.L. (2014). *Chaenomeles speciosa*: A review of chemistry and pharmacology. Biomed. Rep..

[3] Xu R., Kuang M., Li N. (2023). Phytochemistry and pharmacology of plants in the genus Chaenomeles. Arch. Pharm. Res..

[4] Huang W., He J., Nisar M.F., Li H., Wan C. (2018). Phytochemical and Pharmacological Properties of *Chaenomeles speciosa*: An Edible Medicinal Chinese Mugua. Evid. Based Complement. Alternat Med..

[5] Marat N., Danowska-Oziewicz M., Narwojsz A. (2022). *Chaenomeles* Species-Characteristics of Plant, Fruit and Processed Products: A Review. Plants.

[6] Duan Z., Jin C., Deng Y., Liu J., Gu C., Wang J., Cai X., Li S., Zhou Y. (2023). Exploring the chondroprotective effect of *Chaenomeles speciosa* on Glucose-6-Phosphate Isomerase model mice using an integrated approach of network pharmacology and experimental validation. J. Ethnopharmacol..

[7] Zhang L., Cheng Y.X., Liu A.L., Wang H.D., Wang Y.L., Du G.H. (2010). Antioxidant, anti-inflammatory and anti-influenza properties of components from *Chaenomeles speciosa*. Molecules.

[8] The Commission of Chinese Pharmacopoeia (2020). Pharmacopoeia of the People’s Republic of China: Volume 1.

[9] Zhang S., Ren Y., Zhao Q., Wu Y., Zhuo Y., Li H. (2023). Drought-induced CsMYB6 interacts with CsbHLH111 to regulate anthocyanin biosynthesis in *Chaenomeles speciosa*. Physiol. Plant.

[10] Ma Y., Li J., Li J., Yang L., Wu G., Liu S. (2022). Comparative Metabolomics Study of *Chaenomeles speciosa* (Sweet) Nakai from Different Geographical Regions. Foods.

[11] He S., Weng D., Zhang Y., Kong Q., Wang K., Jing N., Li F., Ge Y., Xiong H., Wu L. (2023). A telomere-to-telomere reference genome provides genetic insight into the pentacyclic triterpenoid biosynthesis in *Chaenomeles speciosa*. Hortic. Res..

[12] Fang Q., Zheng H., Fu G., Yin M., Jiang L., Zhao Y., Zha L., Chu S., Peng H., Huang L. (2023). Integrated untargeted metabolome, full-length sequencing, and transcriptome analyses reveal insights into the fruit quality at different harvest times of *Chaenomeles speciosa*. Food Res. Int..

[13] Song Y.L., Zhang L., Gao J.M., Du G.H., Cheng Y.X. (2008). Speciosaperoxide, a new triterpene acid, and other terpenoids from Chaenomeles speciosa. J. Asian Nat. Prod Res..

[14] Wang Z.J., Jin D.N., Zhou Y., Sang X.Y., Zhu Y.Y., He Y.J., Xie T.Z., Dai Z., Zhao Y.L., Luo X.D. (2021). Bioactivity Ingredients of *Chaenomeles speciosa* against Microbes: Characterization by LC-MS and Activity Evaluation. J. Agric. Food Chem..

[15] Jin M., Shi J., Zhu W., Yao H., Wang D.A. (2021). Polysaccharide-Based Biomaterials in Tissue Engineering: A Review. Tissue Eng. Part. B Rev..

[16] Fan Y., Zhou X., Huang G. (2022). Preparation, structure, and properties of tea polysaccharide. Chem. Biol. Drug Des..

[17] Fu J., Li J., Sun Y., Liu S., Song F., Liu Z. (2023). In-depth investigation of the mechanisms of Schisandra chinensis polysaccharide mitigating Alzheimer’s disease rat via gut microbiota and feces metabolomics. Int. J. Biol. Macromol..

[18] Zheng Y., Xie Q., Wang H., Hu Y., Ren B., Li X. (2020). Recent advances in plant polysaccharide-mediated nano drug delivery systems. Int. J. Biol. Macromol..

[19] Eghbaljoo H., Sani I.K., Sani M.A., Rahati S., Mansouri E., Molaee-Aghaee E., Fatourehchi N., Kadi A., Arab A., Sarabandi K. (2022). Advances in plant gum polysaccharides; Sources, techno-functional properties, and applications in the food industry-A review. Int. J. Biol. Macromol..

[20] Canalejo D., Guadalupe Z., Martínez-Lapuente L., Ayestarán B., Pérez-Magariño S. (2021). Optimization of a method to extract polysaccharides from white grape pomace by-products. Food Chem..

[21] Hou C.Y., Yin M.S., Lan P., Wang H.R., Nie H., Ji X.L. (2021). Recent progress in the research of *Angelica sinensis* (Oliv.) Diels polysaccharides: Extraction, purification, structure and bioactivities. Chem. Biol. Technol. Agric..

[22] Zhu Q., Liao C., Liu Y., Wang P., Guo W., He M., Huang Z. (2012). Ethanolic extract and water-soluble polysaccharide from *Chaenomeles speciosa* fruit modulate lipopolysaccharide-induced nitric oxide production in RAW264.7 macrophage cells. J. Ethnopharmacol..

[23] Xie X., Zou G., Li C. (2016). Purification, characterization and in vitro antioxidant activities of polysaccharide from *Chaenomeles speciosa*. Int. J. Biol. Macromol..

[24] Zhou S., Huang G., Huang H. (2022). Extraction, derivatization and antioxidant activities of onion polysaccharide. Food Chem..

[25] Chen F., Huang G., Yang Z., Hou Y. (2019). Antioxidant activity of *Momordica charantia* polysaccharide and its derivatives. Int. J. Biol. Macromol..

[26] Hu W., Yu A., Wang S., Bai Q., Tang H., Yang B., Wang M., Kuang H. (2023). Extraction, Purification, Structural Characteristics, Biological Activities, and Applications of the Polysaccharides from *Zingiber officinale* Roscoe. (Ginger): A Review. Molecules.

[27] Zhan K., Ji X.L., Luo L. (2023). Recent progress in research on *Momordica charantia* polysaccharides: Extraction, purification, structural characteristics and bioactivities. Chem. Biol. Technol. Agric..

[28] Wang Y., Xiong X., Huang G. (2023). Ultrasound-assisted extraction and analysis of maidenhairtree polysaccharides. Ultrason. Sonochem..

[29] Hamed Y.S., Ahsan H.M., Hussain M., Ahmad I., Tian B., Wang J., Zou X.G., Bu T., Ming C., Rayan A.M. (2024). Polysaccharides from *Brassica rapa* root: Extraction, purification, structural features, and biological activities. A review. Int. J. Biol. Macromol..

[30] Kumari N., Kumar M., Radha, Rais N., Puri S., Sharma K., Natta S., Dhumal S., Damale R.D., Kumar S. (2024). Exploring apple pectic polysaccharides: Extraction, characterization, and biological activities-A comprehensive review. Int. J. Biol. Macromol..

[31] Liu G., Ye J., Li W., Zhang J., Wang Q., Zhu X.A., Miao J.Y., Huang Y.H., Chen Y.J., Cao Y. (2020). Extraction, structural characterization, and immunobiological activity of ABP Ia polysaccharide from *Agaricus bisporus*. Int. J. Biol. Macromol..

[32] Chen G., Yuan Q., Saeeduddin M., Ou S., Zeng X., Ye H. (2016). Recent advances in tea polysaccharides: Extraction, purification, physicochemical characterization and bioactivities. Carbohydr. Polym..

[33] Huang Y., Chen H., Zhang K., Lu Y., Wu Q., Chen J., Li Y., Wu Q., Chen Y. (2022). Extraction, purification, structural characterization, and gut microbiota relationship of polysaccharides: A review. Int. J. Biol. Macromol..

[34] Xu J., Zhang J., Sang Y., Wei Y., Chen X., Wang Y., Xue H. (2022). Polysaccharides from Medicine and Food Homology Materials: A Review on Their Extraction, Purification, Structure, and Biological Activities. Molecules.

[35] Lin B., Huang G. (2022). Extraction, isolation, purification, derivatization, bioactivity, structure-activity relationship, and application of polysaccharides from *White jellyfungus*. Biotechnol. Bioeng..

[36] Deng Y., Huang L., Zhang C., Xie P., Cheng J., Wang X., Liu L. (2020). Novel polysaccharide from *Chaenomeles speciosa* seeds: Structural characterization, α-amylase and α-glucosidase inhibitory activity evaluation. Int. J. Biol. Macromol..

[37] Xie X., Zou G., Li C. (2015). Antitumor and immunomodulatory activities of a water-soluble polysaccharide from *Chaenomeles speciosa*. Carbohydr. Polym..

[38] Mazepa E., Biscaia S.M.P., de L Bellan D., da S Trindade E., Simas F.F. (2022). Structural characteristics of native and chemically sulfated polysaccharides from seaweed and their antimelanoma effects. Carbohydr. Polym..

[39] Liu X., Yang L., Li G., Jiang Y., Zhang G., Ling J. (2023). A novel promising neuroprotective agent: *Ganoderma lucidum* polysaccharide. Int. J. Biol. Macromol..

[40] Cheng N., Wang H., Hao H., Rahman F.U., Zhang Y. (2023). Research progress on polysaccharide components of *Cistanche deserticola* as potential pharmaceutical agents. Eur. J. Med. Chem..

[41] Rao Z., Zhou H., Li Q., Zeng N., Wang Q. (2024). Extraction, purification, structural characteristics and biological properties of the polysaccharides from Radix Saposhnikoviae: A review. J. Ethnopharmacol..

[42] Yang X., Yu A., Hu W., Zhang Z., Ruan Y., Kuang H., Wang M. (2023). Extraction, Purification, Structural Characteristics, Health Benefits, and Application of the Polysaccharides from *Lonicera japonica* Thunb.: A Review. Molecules.

[43] Cheng X., Shi S., Su J., Xu Y., Ordaz-Ortiz J.J., Li N., Wu J., Wang H., Wang S. (2020). Structural characterization of a heteropolysaccharide from fruit of *Chaenomelese speciosa* (Sweet) Nakai and its antitumor activity. Carbohydr. Polym..

[44] Liang X., Liu M., Wei Y., Tong L., Guo S., Kang H., Zhang W., Yu Z., Zhang F., Duan J.A. (2023). Structural characteristics and structure-activity relationship of four polysaccharides from *Lycii fructus*. Int. J. Biol. Macromol..

[45] Huang D., Jiang S., Du Z., Chen Y., Xue D., Wang X., Li M., Zhang F., Chen W., Sun L. (2022). Analgesic and Anti-Arthritic Activities of Polysaccharides in *Chaenomeles speciosa*. Front. Pharmacol..

[46] Liu J., Song J., Chen W., Sun L., Zhao Y., Zong Y., He Z., Du R. (2024). Assessment of cytotoxicity, acute, subacute toxicities and antioxidant activities (in vitro) of *Sanghuangporus vaninii* crude polysaccharide. J. Ethnopharmacol..

[47] Hu Z., Wang J., Jin L., Zong T., Duan Y., Sun J., Zhou W., Li G. (2022). Preparation, Characterization and Anti-Complementary Activity of Three Novel Polysaccharides from *Cordyceps militaris*. Polymers.

[48] Sheng Z., Wen L., Yang B. (2022). Structure identification of a polysaccharide in mushroom Lingzhi spore and its immunomodulatory activity. Carbohydr. Polym..

[49] Yu X., Miao Z., Zhang L., Zhu L., Sheng H. (2023). Extraction, purification, structure characteristics, biological activities and pharmaceutical application of Bupleuri Radix Polysaccharide: A review. Int. J. Biol. Macromol..

[50] Harreiter J., Roden M. (2023). Diabetes mellitus– Definition, Klassifikation, Diagnose, Screening und Prävention (Update 2023) [Diabetes mellitus: Definition, classification, diagnosis, screening and prevention (Update 2023)]. Wien Klin Wochenschr..

[51] Majety P., Lozada Orquera F.A., Edem D., Hamdy O. (2023). Pharmacological approaches to the prevention of type 2 diabetes mellitus. Front. Endocrinol..

[52] Fan W., Pang H., Xie Z., Huang G., Zhou Z. (2022). Circular RNAs in diabetes mellitus and its complications. Front. Endocrinol..

[53] Ji X.L., Guo J.H., Cao T.Z., Zhang T.T., Liu Y.Q., Yan Y.Z. (2023). Review on mechanisms and structure-activity relationship of hypoglycemic effects of polysaccharides from natural resources. Food Sci. Hum. Wellness.

[54] Wei J.P., Wang Q.H., Zheng H.J., Wei F. (2018). Research Progress on Non-Drug Treatment for Blood Glucose Control of Type 2 Diabetes Mellitus. Chin. J. Integr. Med..

[55] Fu X., Yang H., Ma C., Li X., Li D., Yang Y., Xu Y., Wang L. (2020). Characterization and inhibitory activities on α-amylase and α-glucosidase of the polysaccharide from blue honeysuckle berries. Int. J. Biol. Macromol..

[56] Mushtaq A., Azam U., Mehreen S., Naseer M.M. (2023). Synthetic α-glucosidase inhibitors as promising anti-diabetic agents: Recent developments and future challenges. Eur. J. Med. Chem..

[57] Martins J.D., Liberal J., Silva A., Ferreira I., Neves B.M., Cruz M.T. (2015). Autophagy and inflammasome interplay. DNA Cell Biol..

[58] Pu W.L., Zhang M.Y., Bai R.Y., Sun L.K., Li W.H., Yu Y.L., Zhang Y., Song L., Wang Z.X., Peng Y.F. (2020). Anti-inflammatory effects of *Rhodiola rosea* L.: A review. Biomed. Pharmacother..

[59] Gravallese E.M., Firestein G.S. (2023). Rheumatoid Arthritis-Common Origins, Divergent Mechanisms. N. Engl. J. Med..

[60] Panigrahy D., Gilligan M.M., Serhan C.N., Kashfi K. (2021). Resolution of inflammation: An organizing principle in biology and medicine. Pharmacol. Ther..

[61] Wang S., Zhou D., Xu Z., Song J., Qian X., Lv X., Luan J. (2019). Anti-tumor Drug Targets Analysis: Current Insight and Future Prospect. Curr. Drug Targets..

[62] Pérez-Herrero E., Fernández-Medarde A. (2015). Advanced targeted therapies in cancer: Drug nanocarriers, the future of chemotherapy. Eur. J. Pharm. Biopharm..

[63] Wang K., Chen Q., Shao Y., Yin S., Liu C., Liu Y., Wang R., Wang T., Qiu Y., Yu H. (2021). Anticancer activities of TCM and their active components against tumor metastasis. Biomed. Pharmacother..

[64] Fan Y., Ma Z., Zhao L., Wang W., Gao M., Jia X., Ouyang H., He J. (2020). Anti-tumor activities and mechanisms of Traditional Chinese medicines formulas: A review. Biomed. Pharmacother..

[65] Roszczyk A., Turło J., Zagożdżon R., Kaleta B. (2022). Immunomodulatory Properties of Polysaccharides from *Lentinula edodes*. Int. J. Mol. Sci..

[66] Li C.X., Liu Y., Zhang Y.Z., Li J.C., Lai J. (2022). Astragalus polysaccharide: A review of its immunomodulatory effect. Arch. Pharm. Res..

[67] Yin M., Zhang Y., Li H. (2019). Advances in Research on Immunoregulation of Macrophages by Plant Polysaccharides. Front. Immunol..

[68] Shapouri-Moghaddam A., Mohammadian S., Vazini H., Taghadosi M., Esmaeili S.A., Mardani F., Seifi B., Mohammadi A., Afshari J.T., Sahebkar A. (2018). Macrophage plasticity, polarization, and function in health and disease. J. Cell Physiol..

[69] Li Z., He X., Liu F., Wang J., Feng J. (2018). A review of polysaccharides from Schisandra chinensis and Schisandra sphenanthera: Properties, functions and applications. Carbohydr. Polym..

[70] Farooqi A.A., Rakhmetova V., Kapanova G., Mussakhanova A., Tashenova G., Tulebayeva A., Akhenbekova A., Xu B. (2023). Suppressive effects of bioactive herbal polysaccharides against different cancers: From mechanisms to translational advancements. Phytomedicine.

[71] Ji X.L., Guo J.H., Tian J.Y., Ma K., Liu Y.Q. (2023). Research progress on degradation methods and product properties of plant polysaccharides. J. Light. Ind..

[72] Fernandes P.A.R., Coimbra M.A. (2023). The antioxidant activity of polysaccharides: A structure-function relationship overview. Carbohydr. Polym..

[73] Ferreira S.S., Passos C.P., Madureira P., Vilanova M., Coimbra M.A. (2015). Structure-function relationships of immunostimulatory polysaccharides: A review. Carbohydr. Polym..

[74] Zhang K., Liu S., Liang S., Xiang F., Wang X., Lian H., Li B., Liu F. (2024). Exopolysaccharides of lactic acid bacteria: Structure, biological activity, structure-activity relationship, and application in the food industry: A review. Int. J. Biol. Macromol..

[75] Tan H.F., Gan C.Y. (2016). Polysaccharide with antioxidant, α-amylase inhibitory and ACE inhibitory activities from *Momordica charantia*. Int. J. Biol. Macromol..

[76] Wang B.H., Cao J.J., Zhang B., Chen H.Q. (2019). Structural characterization, physicochemical properties and α-glucosidase inhibitory activity of polysaccharide from the fruits of wax apple. Carbohydr. Polym..

[77] Hsu W.K., Hsu T.H., Lin F.Y., Cheng Y.K., Yang J.P. (2013). Separation, purification, and α-glucosidase inhibition of polysaccharides from *Coriolus versicolor* LH1 mycelia. Carbohydr. Polym..

[78] Jia Y., Xue Z., Wang Y., Lu Y., Li R., Li N., Wang Q., Zhang M., Chen H. (2021). Chemical structure and inhibition on α-glucosidase of polysaccharides from corn silk by fractional precipitation. Carbohydr. Polym..

[79] Wang J., Dai G., Shang M., Wang Y., Xia C., Duan B., Xu L. (2023). Extraction, structural-activity relationships, bioactivities, and application prospects of *Pueraria lobata* polysaccharides as ingredients for functional products: A review. Int. J. Biol. Macromol..

[80] Shen W., Chen C., Guan Y., Song X., Jin Y., Wang J., Hu Y., Xin T., Jiang Q., Zhong L. (2017). A pumpkin polysaccharide induces apoptosis by inhibiting the JAK2/STAT3 pathway in human hepatoma HepG2 cells. Int. J. Biol. Macromol..

[81] Tian Y., Zhao Y., Zeng H., Zhang Y., Zheng B. (2016). Structural characterization of a novel neutral polysaccharide from *Lentinus giganteus* and its antitumor activity through inducing apoptosis. Carbohydr. Polym..

[82] Joseph M.M., Aravind S.R., George S.K., Varghese S., Sreelekha T.T. (2013). A galactomannan polysaccharide from *Punica granatum* imparts in vitro and in vivo anticancer activity. Carbohydr. Polym..

[83] Bvenura C., Sivakumar D. (2017). The role of wild fruits and vegetables in delivering a balanced and healthy diet. Food Res. Int..

[84] Slavin J.L., Lloyd B. (2012). Health benefits of fruits and vegetables. Adv. Nutr..

[85] Tao W., Zhao C., Lin G., Wang Q., Lv Q., Wang S., Chen Y. (2022). UPLC-ESI-QTOF-MS/MS Analysis of the Phytochemical Compositions From *Chaenomeles speciosa* (Sweet) Nakai Fruits. J. Chromatogr. Sci..

[86] Du H., Wu J., Li H., Zhong P.X., Xu Y.J., Li C.H., Ji K.X., Wang L.S. (2013). Polyphenols and triterpenes from Chaenomeles fruits: Chemical analysis and antioxidant activities assessment. Food Chem..

[87] Qin D., Han S., Liu M., Guo T., Hu Z., Zhou Y., Luo F. (2023). Polysaccharides from Phellinus linteus: A systematic review of their extractions, purifications, structures and functions. Int. J. Biol. Macromol..

[88] Xu R., Deng P., Ma Y., Li K., Ren F., Li N. (2024). Anti-Hyperuricemic Effects of Extracts from *Chaenomeles speciosa* (Sweet) Nakai Fruits on Hyperuricemic Rats. Metabolites.

[89] Zhaogao L., Yaxuan W., Mengwei X., Haiyu L., Lin L., Delin X. (2023). Molecular mechanism overview of metabolite biosynthesis in medicinal plants. Plant Physiol. Biochem..

[90] Kumar M., Hasan M., Sharma A., Suhag R., Maheshwari C., Radha, Chandran D., Sharma K., Dhumal S., Senapathy M. (2023). *Tinospora cordifolia* (Willd.) Hook.f. & Thomson polysaccharides: A review on extraction, characterization, and bioactivities. Int. J. Biol. Macromol..

[91] Chen X., Liu Y., Ren L., Dai X., Zhao J., Gao C., Zhang S., Dong J., Zhao Z., Li Y. (2024). Extraction, purification, structural characteristics and biological properties of the polysaccharides from *Armillaria mellea* (Vahl) P. Kumm.: A review. Int. J. Biol. Macromol..

[92] Meghwanshi G.K., Kaur N., Verma S., Dabi N.K., Vashishtha A., Charan P.D., Purohit P., Bhandari H.S., Bhojak N., Kumar R. (2020). Enzymes for pharmaceutical and therapeutic applications. Biotechnol. Appl. Biochem..

[93] Chen P., Xu Y., Yang S., Chang Q., Zheng B., Zhang Y., Hu X., Zeng H. (2021). Application of X-ray diffraction and energy dispersive spectroscopy in the isolation of sulfated polysaccharide from *Porphyra haitanensis* and its antioxidant capacity under in vitro digestion. J. Sci. Food Agric..

[94] Zdunek A., Pieczywek P.M., Cybulska J. (2021). The primary, secondary, and structures of higher levels of pectin polysaccharides. Compr. Rev. Food Sci. Food Saf..

